# Influence of Shear-Induced Pre-Crosslinking on the Mechanical and Dielectric Properties of Crosslinked Polyethylene Cable Insulation

**DOI:** 10.3390/ma19061216

**Published:** 2026-03-19

**Authors:** Mingjie Jiang, Xuan Wang, Runsheng Zhang, Zilin Tian

**Affiliations:** State Key Laboratory of High-Efficiency Special Cable Technology, Harbin University of Science and Technology, Harbin 150080, China; 2420300042@stu.hrbust.edu.cn (M.J.); zrs2583148@163.com (R.Z.); 2310303054@stu.hrbust.edu.cn (Z.T.)

**Keywords:** crosslinked polyethylene, shear effect, pre-crosslinking, insulation properties, extrusion process

## Abstract

**Highlights:**

- Pre-crosslinking reduces XLPE crosslinking degree.
- Mechanical properties decline with pre-crosslinking but still meet industry standards.
- Dielectric performance deteriorates: higher conduction current; lower breakdown strength.
- The experiment results show that the performance of XLPE cable insulation material can be improved by reducing the pre-crosslinking.
- The higher pre-crosslinking temperature can reduce but not eliminate the performance degradation.
- The results provide key insights for optimizing the extrusion process of high-voltage cables to suppress pre-crosslinking.

**Abstract:**

Crosslinked polyethylene (XLPE) is a widely used cable insulation material for power cables at various voltage levels, offering excellent electrical, mechanical, and thermal stability. However, during the continuous extrusion moulding process, prolonged shear action and localized temperature accumulation can easily induce premature crosslinking. This leads to a decline in melt rheological properties and reduced processing stability, as well as having an adverse effect on the microstructure and overall performance of the formed insulation layer. This study systematically investigated the impact of shear-induced pre-crosslinking on the mechanical properties and dielectric characteristics of XLPE cable insulation materials through experimental testing methods. The experimental results demonstrate that, while premature crosslinking has a minimal effect on mechanical properties, it significantly deteriorates dielectric performance, as evidenced by increased conduction current, reduced breakdown strength, and compromised microstructural integrity. These findings suggest that, to improve the quality and reliability of XLPE cable production, engineering designs should prioritize controlling the pre-crosslinking process to ensure stable dielectric performance.

## 1. Introduction

Crosslinked polyethylene (XLPE) is the preferred material for power cable insulation, occupying a dominant position in global power transmission networks thanks to its excellent electrical insulation properties, thermal stability, and mechanical strength [1]. The accelerated construction of ultra-high voltage and smart grids means that cable insulation materials must meet more stringent requirements regarding purity, homogeneity, and long-term operational reliability. Once a three-dimensional network structure has been formed through chemical crosslinking, polyethylene retains its original excellent electrical and processing properties while significantly enhancing thermal stability, mechanical strength, and anti-creep capability. This enables it to maintain structural stability at operating temperatures above 90 °C in the long term, meeting the requirements of modern power systems for high-capacity transmission [2,3,4]. Manufacturing cable insulation layers mainly relies on continuous extrusion moulding processes involving complex thermal field changes and intense shear effects exerted by the screws on the melt.

The extrusion process is a key link in cable insulation production and has a crucial impact on cable quality and performance. Currently, ultra-high voltage submarine cables are much longer than land cables, with individual cables often exceeding 20 km in length and requiring continuous extrusion production for over 15 days. During this process, XLPE insulation materials may undergo pre-crosslinking due to shear effects, thereby affecting cable quality [5,6].

The peroxide-initiated crosslinking of XLPE (crosslinked polyethylene) is a widely used radical reaction process in the production of power cable insulation materials. The most commonly used crosslinking agent in this process is diisopropylbenzene peroxide (DCP), which undergoes thermal decomposition at temperatures of around 160–180 °C. This results in the homolytic cleavage of O-O bonds and the generation of highly reactive free radicals. These free radicals then abstract hydrogen atoms from the polyethylene chains to form large molecular alkyl radicals. These large molecular radicals then combine to form covalent carbon–carbon bonds, linking independent polyethylene chains and constructing a three-dimensional network structure. This process significantly enhances the mechanical strength, heat resistance, and electrical insulation properties of polyethylene [7,8,9]. The crosslinking temperature and time are key parameters in the preparation of XLPE and significantly influence its crystallization behaviour and dielectric breakdown performance [10,11,12].

Currently, most existing research focuses on the rheological behaviour of polyethylene in an uncrosslinked state, or on the ageing and breakdown characteristics of fully crosslinked XLPE material systems [13,14,15,16]. There is a lack of systematic experimental data and theoretical support for material systems in an intermediate state involving partial crosslinking under shear forces, followed by full crosslinking at a high temperature [17,18]. In particular, the impact of pre-crosslinking on the dielectric properties of materials remains largely qualitative. Ren Dongxue et al. [19] found that the greater the content of terminal vinyl groups in LDPE, the more unstable the XLPE becomes during extrusion and heating crosslinking, resulting in faster crosslinking reactions, increased gel content, and higher complex viscosity. Wang et al. [20] demonstrated that increasing the crosslinking temperature appropriately can enhance the gel content and mechanical properties of XLPE; however, excessively high temperatures lead to over-crosslinking, which degrades performance. Zhou et al. [21,22] found that, as the degree of crosslinking increases, XLPE forms a dense three-dimensional network structure that significantly improves breakdown strength and insulation properties. Current experiments on XLPE insulation material properties mostly use one-step crosslinking methods. However, in actual XLPE cable production, the insulation material undergoes two steps: low-temperature pre-crosslinking and high-temperature crosslinking. However, insufficient experimentation has been conducted to study the impact of a pre-crosslinking process on XLPE insulation properties [23,24].

Pre-crosslinking can negatively impact XLPE cable manufacturing and operation [25,26,27]. Pre-crosslinking structures act as internal defects in the insulation, causing microstructural inhomogeneity and generating stress concentration areas. This affects the cable’s mechanical and dielectric properties and reduces long-term operational reliability. Based on our research findings, the pre-crosslinking process has a relatively minor effect on the mechanical properties of polymer materials, but a significant impact on their dielectric properties, particularly their breakdown performance. This phenomenon has been extensively studied and confirmed in various insulating polymer materials. Pre-crosslinking behaviour involves forming a pre-established crosslinking network, which has a profound influence on the material’s electrical characteristics.

Therefore, a thorough clarification of the influence of pre-crosslinking on material properties is important for optimizing extrusion processes, developing high-performance XLPE insulation materials, and improving the quality of cable manufacturing. This paper aims to address the issue of shear-induced pre-crosslinking of XLPE cable insulation materials during long-term extrusion and the limitations of existing research. It focuses on the impact of pre-crosslinking on XLPE performance. Two-step crosslinking was used to prepare XLPE samples with different levels of pre-crosslinking (at 140, 150, and 160 °C) to simulate the behaviour that may occur during long-term extrusion. Normally crosslinked XLPE samples were prepared as a control group. The impact of pre-crosslinking on the microstructure of XLPE was studied through gel content and differential scanning calorimetry (DSC) testing. The impact on mechanical properties was studied through stress–strain and thermal extension testing, while the impact on dielectric properties was studied through conductivity, current, breakdown field strength, and dielectric spectrum testing. This study focuses on analyzing the degradation mechanism of pre-crosslinking on DC conductivity characteristics and breakdown field strength. This provides a theoretical basis for optimizing the extrusion process parameters for XLPE cables of higher voltage grades, as well as providing a theoretical basis and experimental support for suppressing pre-crosslinking phenomena.

## 2. Materials and Methods

### 2.1. Experimental Materials

The study used low-density polyethylene (LDPE) as the base resin, specifically LD200GH produced by Sinopec Beijing Yanhua Company (Beijing, China) with a melt index (MI) of 2.0 g/10 min. The crosslinking agent used was dicumyl peroxide (DCP, ≥99% purity), supplied by Shanghai Gaoqiao Chemical Co., Ltd., Shanghai, China. Antioxidant 1010 was obtained from Tianjin Lisheng Chemical Co., Ltd., Tianjin, China. All materials were dried at 80 °C in a vacuum oven for 24 h prior to use to eliminate the effects of moisture.

### 2.2. Experimental Equipment

The experimental setup incorporated several key pieces of equipment for processing and characterizing materials. An RM-200A torque rheometer, manufactured by Harbin Hapu Electric Technology Company (Harbin, China), was used to analyze the kinetics of the crosslinking reaction. Crosslinking and moulding of samples were performed using an LB25D compression moulding machine supplied by Huzhou Shuangli Automation Technology Equipment Company (Huzhou, China). Material drying and specimen pretreatment were conducted in a DZF-6090 vacuum drying oven produced by the Shanghai Yiheng Scientific Instrument Company (Shanghai, China). Tensile testing to evaluate mechanical properties was carried out on a CMT6000 universal testing machine from MTS Industrial Systems (Shenzhen, China). Electrical characterization involved two specialized instruments: a C60/3 high-voltage tester, which was used to measure AC/DC breakdown strength and was supplied by Suzhou Huadian Electric Company (Suzhou, China), and a DMJ-450 vacuum coating system, which was provided by Beijing Zhongke Technology Development Company (Beijing, China).

### 2.3. Sample Preparation

Preparation of uncross-linked insulating material: The temperature of the mixing chamber in the torque rheometer was set to 110 °C and the rotor speed was adjusted to 60 rpm. Once the temperature had stabilized, 40 g of LDPE pellets were weighed and added to the mixer. The mixture was then blended for 4 min until the torque stabilized, which indicated that the material had completely melted. Then, 0.3 phr of antioxidant 1010 was added and mixed for an additional 5 min to ensure uniform dispersion. Finally, 1.8 phr of crosslinking agent DCP was introduced and mixed for three minutes before the material was removed, yielding the uncross-linked insulating material.

Preparation of pre-crosslinked samples: A two-step crosslinking method was employed for this purpose. For the first step (pre-crosslinking), the pre-crosslinking behaviour during extrusion was simulated at lower temperatures (e.g., 120–125 °C) and under shear stress. The temperature of the torque rheometer was set to 140, 150, and 160 °C, respectively, while the rotor speed was maintained at 30 rpm. Once the temperature had stabilized, the uncrosslinked insulating material was placed in the mixer. The reaction time was recorded once the torque began to rise, and the sample was removed after 150 s of pre-crosslinking. In the second step (high-temperature crosslinking), final crosslinking was completed at temperatures of 180–200 °C to form a three-dimensional network structure. The pre-crosslinked samples were placed in a plate vulcanising press and crosslinked at 180 °C under 15 MPa of pressure for 600 s. After crosslinking, the samples were allowed to cool naturally to room temperature.

Preparation of control group samples: The uncrosslinked insulating material was placed directly into the plate vulcanising press and crosslinked at 180 °C under 15 MPa of pressure for 600 s. After cooling to room temperature, the normally cross-linked samples were obtained for use as the control group in performance comparisons.

All samples were then placed in a vacuum drying oven at 80 °C for 24 h to degas and dry, and to remove by-products generated during the crosslinking reaction, thus avoiding interference with subsequent performance tests. Depending on the testing requirements, the samples were processed into the following dimensions:

- Gel content test samples: Square sheets measuring 10 mm × 10 mm × 0.2 mm.
- Tensile and hot elongation test samples: Square sheets measuring 10 mm × 10 mm × 1 mm.
- Conductivity test samples: Square sheets measuring 10 mm × 10 mm × 0.2 mm.
- Breakdown strength test samples: Circular sheets measuring 8 mm in diameter and 0.05 mm in thickness.

### 2.4. Test Methods

#### 2.4.1. Material Characterization Experiment

Crosslinking degree test: The crosslinking degree of XLPE was characterized using the gel content method in accordance with the ISO 18437-1:2012 standard [28]. The detailed procedure is as follows: A stainless steel mesh was cut into 20 mm × 20 mm square pouches, and the weight was recorded as *M_1_*. Approximately 0.5 g of the sample was weighed and cut into small pieces before being placed in the stainless steel mesh pouch. The weight was recorded as *M*_2_. The pouch containing the sample was fixed in a three-neck flask and immersed completely in xylene solvent. The flask was then refluxed at 150 °C for 12 h. After extraction, the pouch was placed in a vacuum oven at 80 °C for 12 h, then cooled to room temperature and weighed (*M*_3_). The gel content (*G*) was calculated using the following formula: *G =* (*M*_3_ − *M*_1_)/(*M*_2_ − *M*_1_) × 100%. Three parallel tests were conducted for each sample group, and the data from the three experiments were statistically analyzed.

Differential scanning calorimetry test: DSC was used to analyze the crystallization and melting behaviour of XLPE samples. Measurements were performed using a DSC instrument under a nitrogen atmosphere to prevent oxidative degradation. Approximately 5 ± 0.5 mg of each sample was sealed in an aluminum pan and first heated from room temperature to 150 °C to eliminate thermal history. This was followed by cooling to 25 °C and reheating to 150 °C. The heating and cooling rates were both set at 10 °C/min and the nitrogen flow rate was maintained at 150 mL/min. The crystallinity *X_c_* of the sample was calculated using the following formula:

(1) $$X_{c}=\;\frac{{∆H}_{m}}{{∆H}_{100}}\;\times 100\%$$

where Δ*H_m_* represents the melting enthalpy obtained from the area of the melting peak in the DSC curve and Δ*H*_100_ is the melting enthalpy of 100% crystalline polyethylene, taken as 287.3 J/g.

#### 2.4.2. Mechanical Performance Tests

**Figure 1:**
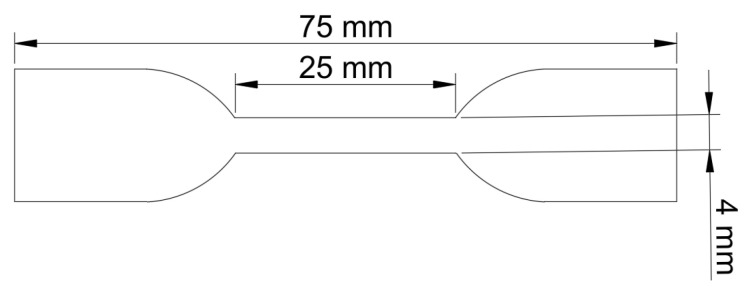
Schematic diagram of sample size.

Hot Set Test: The samples were cut into standard dumbbell shapes (as shown in Figure 1), and a 25 mm reference line was marked at the narrow section. According to the requirements of IEC60811-1-1:2001 [29], the sample was vertically fixed on a test frame during testing, with a corresponding load (load density: 0.2 N/mm^2^) suspended at the lower end. The sample was then placed in a 200 °C oven for 15 min, and the length of the marked line *L*_1_ was measured. Afterward, the sample was cut, kept in the oven for another 5 min, cooled to room temperature, and the marked line length *L*_2_ was measured. The hot elongation rate and permanent deformation rate were calculated as:

(2) $$\mathrm{Load}\;\mathrm{elongation}\;\mathrm{rate}=\;\frac{\left(L_{1}\;-\;L_{0}\right)}{L_{0}}\;\times 100\%$$

(3) $$\mathrm{Permanent}\;\mathrm{deformation}\;\mathrm{rate}=\frac{\left(L_{2}-L_{0}\right)}{L_{0}}\times 100\%$$

where *L*_0_ is the initial marked length (25 mm). Each sample group was tested five times in parallel, and the data from the five experiments were statistically analyzed.

Tensile Test: The test was performed using a CMT6000 electronic tensile testing machine and in accordance with the requirements of ISO 527-2:1993 Standard [30] dumbbell-shaped (as shown in Figure 1) specimens were used with a tensile rate of 50 mm/min at room temperature (25 °C). The stress–strain curves were recorded to obtain the tensile strength and elongation at break. Each sample group underwent five parallel tests, with the average value calculated and the middle set of data selected as the test result for that group [9].

#### 2.4.3. Dielectric Performance Tests

Conduction current test: A three-electrode test system was used with samples measuring 200 ± 5 μm in thickness. Prior to testing, aluminum electrodes were evaporated on both sides of the sample (high-voltage electrode diameter: 75 mm; measuring electrode diameter: 50 mm; guard electrode inner diameter: 55 mm; guard electrode outer diameter: 75 mm). Tests were conducted at room temperature under electric field strengths ranging from 5 kV/mm to 40 kV/mm. After each voltage increase, the system was stabilized for 15 min to eliminate the effects of polarization current, after which the conduction current was recorded. The conduction current density *J* was calculated as *J* = *I*/*S*, where *I* is the conduction current and *S* is the measuring electrode area.

AC/DC breakdown test: A C60/3 high-voltage tester, which was used to measure AC/DC breakdown strength and was supplied by Suzhou Huadian Electric Company (Suzhou, China), was used in accordance with the IEC 60243-1:2013 standard [31]. Samples with a thickness of 50 ± 5 µm were fully immersed in dimethyl silicone oil to prevent partial discharge. For DC breakdown tests, a voltage ramp rate of 1 kV/s was applied; for AC breakdown tests, a voltage at a frequency of 50 Hz was applied at the same ramp rate. The breakdown voltage was recorded, and the breakdown field strength (*E*) was calculated using the formula *E* = *U*/*d*, where *U* is the breakdown voltage and d is the sample thickness. Ten parallel tests were performed for each sample group, and the data were analyzed using a two-parameter Weibull distribution with the characteristic breakdown field strength taken at a cumulative failure probability of 63.2%.

## 3. Results

### 3.1. Characterization and Microstructure of Pre-Crosslinked Materials

#### 3.1.1. Gel Content Results Analysis

**Figure 2:**
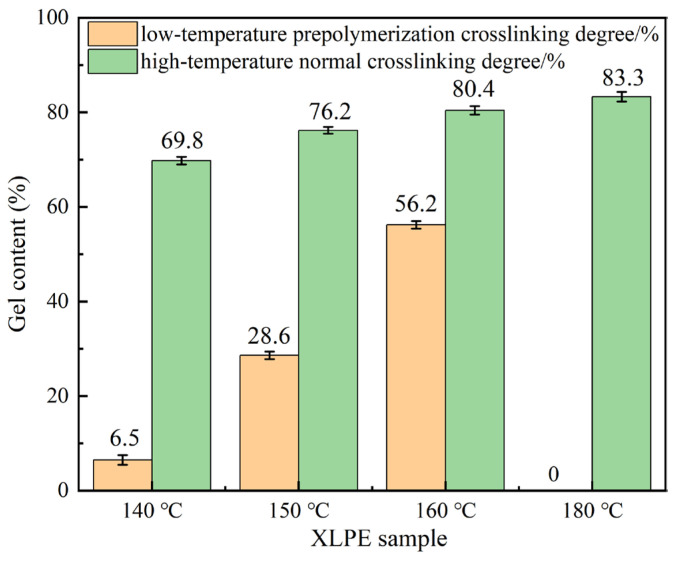
Gel content of XLPE samples.

The degree of crosslinking is a critical indicator of the compactness of the three-dimensional network structure of XLPE and directly governs the material’s mechanical and dielectric properties [32]. Figure 2 presents the gel content test results for various XLPE specimens, obtained through gel content measurement.

As can be seen in Figure 2, the crosslinking degree of the pre-crosslinked samples improves significantly after high-temperature normal crosslinking. However, it remains lower than that of the normally crosslinked XLPE samples. The crosslinking degree of the sample pre-crosslinked at 140 °C is the lowest, at 69.8%, which is 16.2% lower than that of the sample crosslinked at normal temperatures. The crosslinking degrees of the samples pre-crosslinked at 150 °C and 160 °C are 76.2% and 80.4%, respectively; these values are 8.5% and 3.5% lower than that of the normally crosslinked sample. This is because the pre-crosslinking process causes the premature crosslinking of the XLPE sample, and crosslinked structures form inside the sample. These cross-linked structures reduce the mobility of the PE molecular chains during subsequent high-temperature normal crosslinking, which inhibits the formation of a perfect network structure and results in a reduced crosslinking degree.

#### 3.1.2. Analysis of the Behaviour of Crystallization

**Figure 3:**
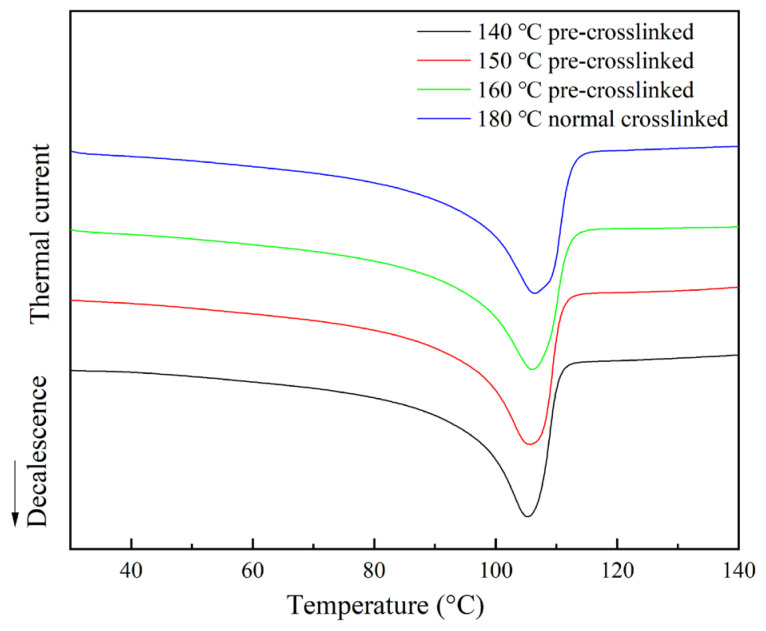
Melt curve of XLPE sample.

To investigate the effect of pre-crosslinking on the microstructure of XLPE further, DSC analysis was used to examine the crystallization and melting behaviour of the samples. Figure 3 shows the DSC curves of the different XLPE specimens, and Table 1 summarizes the corresponding crystallization parameters.

As can be seen from the DSC curves, all XLPE samples exhibit distinct crystallization and melting peaks during cooling and heating, respectively, corresponding to the crystallization and melting processes of polyethylene crystals. Compared with the normally crosslinked sample, the crystallization and melting temperatures of the pre-crosslinked samples are slightly lower, and the crystallinity is also lower. The normally crosslinked sample has a crystallinity of about 36.52%, whereas the pre-crosslinked samples have a crystallinity ranging from approximately 32.85% to 33.15%. Although the differences between the pre-crosslinked samples are relatively small, a slight downward trend in crystallinity can be seen as the pre-crosslinking temperature increases.

XLPE is a typical semi-crystalline polymer, and its crystalline morphology is strongly affected by its thermal history. The pre-crosslinking process alters the material’s thermal history and introduces partial crosslinked structures before the final crosslinking stage. These early-formed crosslinking points restrict the mobility and regular folding of polyethylene molecular chains during the subsequent high-temperature crosslinking process, thereby hindering crystal nucleation and growth. Consequently, the material’s crystallization ability decreases, resulting in lower overall crystallinity in the pre-crosslinked samples compared to the normally crosslinked sample.

However, the differences in crystallinity among the pre-crosslinked samples are relatively small, indicating that the influence of the pre-crosslinking temperature on crystallization behaviour is minimal.

### 3.2. Effect of Pre-Crosslinking on Mechanical Properties

#### 3.2.1. Thermal Extension Properties Analysis

**Figure 4:**
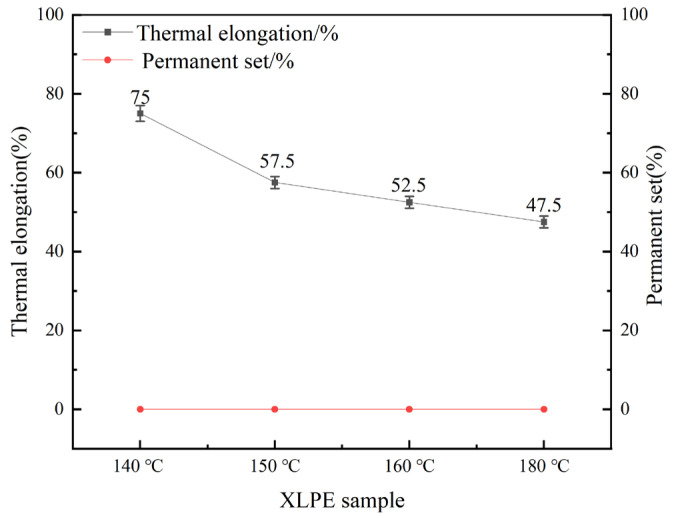
Thermal elongation and permanent deformation of XLPE specimens.

The thermal extension property is a critical indicator of the anti-deformation capability of XLPE materials at high temperatures. This is directly related to the structural stability of cables during high-temperature operation. Figure 4 shows the hot extension and permanent deformation rates of XLPE samples with different pre-crosslinking degrees. As the pre-crosslinking temperature increases, the hot extension rate of the samples gradually decreases. The sample pre-crosslinked at 140 °C exhibits the highest hot extension rate of 75%. The hot extension rates for samples pre-crosslinked at 150 °C and 160 °C are 57.5% and 52.5%, respectively. The normally crosslinked sample shows the lowest hot extension rate of 47.5%. The permanent deformation rate for all samples is 0%, satisfying the ISO 272:1982 standard [33] requirement that the permanent deformation rate does not exceed 15%. The hot extension rates are all below the standard upper limit of 175%.

The thermal elongation rate is inversely related to the degree of crosslinking. Higher crosslinking degrees result in increased intermolecular chain crosslinking density, enhancing resistance to molecular chain slippage at elevated temperatures and consequently reducing thermal elongation rates. The pre-crosslinked sample achieved the highest thermal elongation rate of 75% at 140 °C, while the conventionally crosslinked sample demonstrated the lowest rate of 47.5%. All samples exhibited zero permanent deformation. Conversely, although the conventionally crosslinked samples achieved the highest crosslinking degrees, they exhibited the lowest thermal elongation rates. These test results align with the findings of the crosslinking degree analysis, further confirming the impact of pre-crosslinking on XLPE crosslinking degrees.

#### 3.2.2. Tensile Performance Analysis

**Figure 5:**
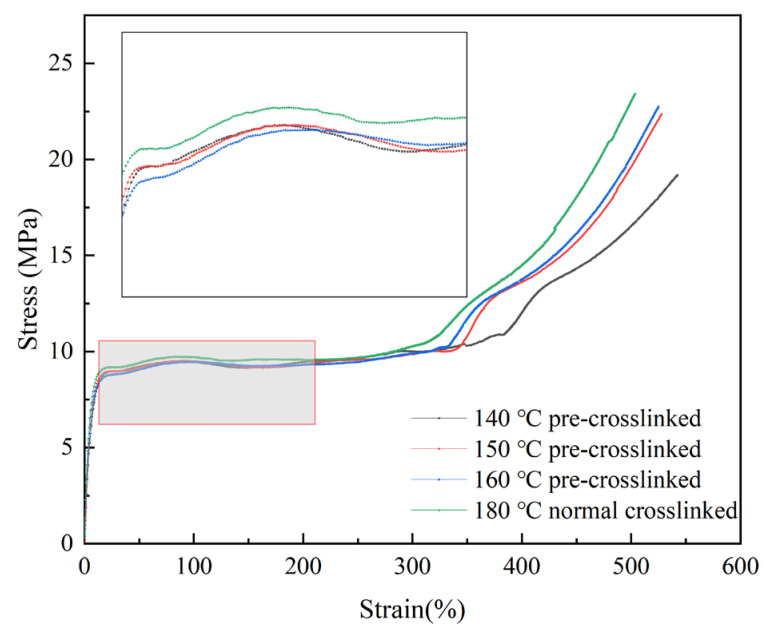
Stress–strain curves of XLPE specimens.

Tensile properties are core indicators of the mechanical performance of XLPE materials, reflecting their ability to resist tensile failure under external forces [34]. Figure 5 shows the stress–strain curves of different XLPE samples, and Table 2 lists the relevant tensile strength and elongation at break parameters. The normally crosslinked sample exhibits the highest tensile strength of 24.54 MPa and the lowest elongation at break of 506.48%. The pre-crosslinked samples all demonstrate lower tensile strengths and higher elongations at break than the normally crosslinked sample. Furthermore, as the pre-crosslinking temperature increases, the tensile strength increases gradually while the elongation at break decreases gradually. The sample pre-crosslinked at 140 °C has the lowest tensile strength of 20.08 MPa and the highest elongation at break of 551.05%. The sample pre-crosslinked at 160 °C has a tensile strength of 22.77 MPa and an elongation at break of 515.05%, properties that are closer to those of the normally crosslinked sample.

The trend in tensile strength variation is closely related to the degree of crosslinking: as the pre-crosslinking temperature increases, the degree of crosslinking in the samples gradually increases, and the crosslinking network structure becomes more complete. This enhances the interaction force between the molecular chains and promotes their entanglement, thereby improving the material’s ability to resist tensile failure and increasing its tensile strength. Conversely, the change in elongation at break is related to the crosslinking point density: the greater the density, the more restricted the relative movement of the molecular chains becomes, resulting in weaker plastic deformation capacity and a lower elongation at break. All samples exceed the lower limit of 17 MPa specified in ISO 527-2:1993 [30] for tensile strength, and the elongation at break exceeds the requirement of 500%. This indicates that, although pre-crosslinking may reduce the tensile strength of XLPE, the material’s mechanical properties still meet practical application requirements.

### 3.3. Effect of Pre-Crosslinking on Dielectric Properties

#### 3.3.1. Conductivity Characteristics Analysis

**Figure 6:**
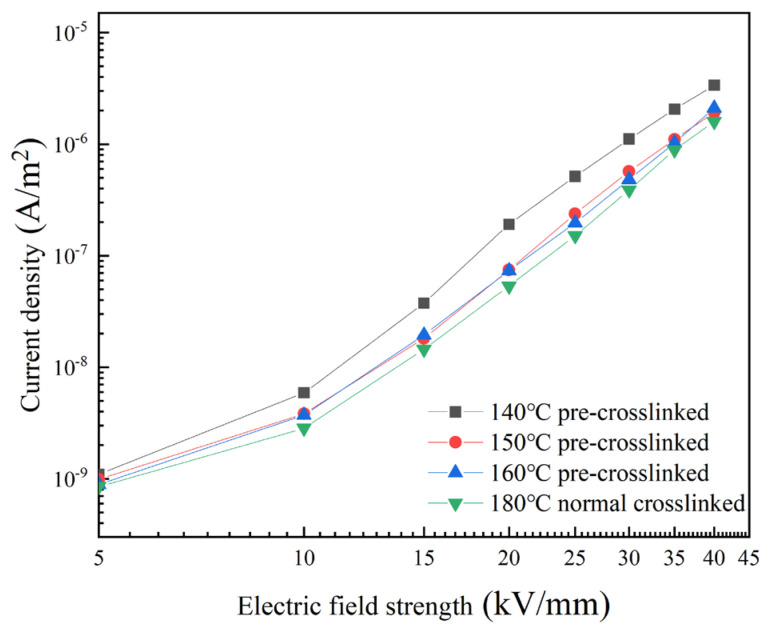
The electrical conductivity density of different XLPE samples.

Conduction current is a key parameter that reflects the dielectric properties of XLPE, and its magnitude is closely related to the migration capability of carriers within the material [35]. Figure 6 shows how conduction current density varies with electric field strength for different XLPE samples. As the electric field strength increases, the conduction current density of all samples gradually increases, in accordance with the fundamental laws of dielectric conduction. The conduction current densities of the pre-crosslinked samples are higher than that of the normally crosslinked sample. The sample pre-crosslinked at 140 °C exhibits the highest conduction current density. The samples pre-crosslinked at 150 °C and 160 °C have similar conduction current densities, which are slightly higher than that of the normally crosslinked sample. At an electric field strength of 40 kV/mm, the conduction current density of the sample pre-crosslinked at 140 °C is approximately 1.2 × 10^−4^ A/m^2^, while that of the normally crosslinked sample is approximately 8.5 × 10^−5^ A/m^2^. The former is 1.4 times greater than the latter.

This phenomenon can be attributed to the pre-crosslinking process reducing the crosslinking degree and decreasing the integrity of the microstructure. This reduces the number of deep traps and increases the proportion of shallow traps, thereby weakening the traps’ ability to capture carriers and increasing their mobility. Ultimately, this results in a higher conduction current density. The sample pre-crosslinked at 140 °C has the lowest crosslinking degree and thus minimal resistance to carrier migration, resulting in the highest conduction current density. As the pre-crosslinking temperature increases, the crosslinking structure becomes progressively more complete, the number of deep traps increases, and carrier migration is more effectively suppressed, causing the conduction current density to gradually decrease, although it remains higher than that of the sample that has undergone normal crosslinking.

#### 3.3.2. AC/DC Breakdown Characteristics Analysis

**Figure 7:**
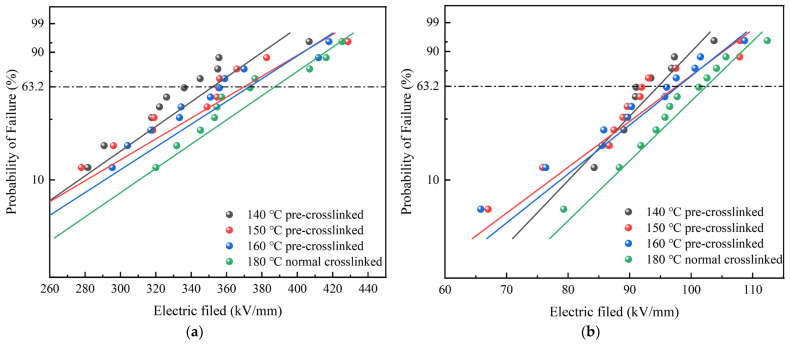
Weibull distribution of AC/DC breakdown field strengths of different XLPE specimens; (**a**) DC breakdown, (**b**) AC breakdown.

Breakdown field strength is the key indicator used to measure the insulating performance of XLPE, reflecting the maximum electric field strength that the material can withstand [36,37,38]. The two-parameter Weibull distribution was used to analyze the AC/DC breakdown test data, producing Weibull distribution plots for the breakdown strength of various XLPE samples, as illustrated in Figure 7. The characteristic breakdown strengths of XLPE samples with different degrees of pre-crosslinking are presented in Table 3.

As shown in Table 3, the normal crosslinked sample exhibits the highest breakdown field strength under both DC and AC conditions. Its characteristic DC and AC breakdown strengths are 378.9 kV/mm and 101.2 kV/mm, respectively. The field strength of the pre-crosslinked samples is lower than that of the normally crosslinked samples. The field strength increases gradually as the pre-crosslinking temperature increases, and the decrease in field strength gradually reduces. The sample pre-crosslinked at 140 °C shows the greatest reduction, with a 9.4% decrease in DC breakdown strength and a 7.6% decrease in AC breakdown strength compared to the normally crosslinked sample. The sample pre-crosslinked at 160 °C shows the smallest reduction, with a 4.2% decrease in DC breakdown strength and a 1.6% decrease in AC breakdown strength.

There are two main reasons for the reduction in breakdown strength caused by pre-crosslinking. Firstly, pre-crosslinking reduces the degree of crosslinking of the sample, resulting in a less dense three-dimensional network structure that cannot effectively block carrier migration. This makes it easier for conductive channels to form. Secondly, during the pre-crosslinking process, the crosslinking agent does not decompose completely. The residual crosslinking agent acts as impurity molecules that dissociate to generate a large number of charges under an electric field. This increases the carrier concentration and reduces the breakdown strength. As the pre-crosslinking temperature increases, the crosslinking degree of the sample gradually increases, the microstructure becomes more complete, and the influence of the above-mentioned factors gradually weakens. Consequently, the breakdown strength gradually increases.

## 4. Conclusions

This study used a two-step crosslinking process to create XLPE samples with different levels of pre-crosslinking. The impact of pre-crosslinking on the physical, mechanical, and dielectric properties of XLPE was analyzed. Key findings are as follows.

Gel content analysis revealed that the degree of crosslinking reduction in XLPE samples pre-crosslinked at different temperatures varied after normal crosslinking. DSC results showed that pre-crosslinked samples had significantly lower crystallinity than normally crosslinked samples, though there were minimal differences in crystallinity among pre-crosslinked samples. Thermal elongation and stress–strain tests showed that pre-crosslinking affected the mechanical properties of the XLPE samples, specifically increasing thermal elongation while reducing tensile strength. However, these effects were not statistically significant, and the material’s performance still met applicable standards.

Pre-crosslinking leads to an increase in conduction current density and a decrease in XLPE’s breakdown field strength. The DC breakdown strength of a sample pre-crosslinked at 140 °C decreased by 9.4%, while the AC breakdown strength decreased by 7.6%, compared to a normally crosslinked sample. As the pre-crosslinking temperature increased, the conduction current density gradually decreased, and the breakdown field strength gradually increased, although it remained lower than that of a normally crosslinked sample.

This research was initially driven by the desire to examine the behaviour of XLPE prior to crosslinking during the cable extrusion process. This behaviour may be influenced by factors such as shear rate, residence time, and local temperature accumulation within the screw channel. These factors can cause crosslinking reactions to occur prematurely, before the designed crosslinking stage. This study aims to evaluate the influence of such pre-crosslinking behaviour on the mechanical and dielectric properties of cable insulation materials. The experimental results demonstrate that pre-crosslinking has a significant impact on XLPE performance, particularly its dielectric properties. These findings provide actionable guidance for industrial production conditions, enabling the following optimisations in practical engineering: (1) maintain the reaction barrel temperature below the peroxide decomposition threshold prior to the designed crosslinking stage; (2) minimize residence time by reducing the retention zone in the screw structure; (3) enhance temperature uniformity to prevent localized heat accumulation; (4) establish safer pre-crosslinking temperature limits for process control.

This study focuses primarily on the impact of pre-crosslinking behaviour on the mechanical and dielectric properties of XLPE insulation materials subjected to shear. In practical applications, fillers are often introduced into polyethylene insulation in order to modify its properties. However, the interaction between fillers and the crosslinked network may also affect the material’s mechanical and dielectric performance. While this aspect was not addressed in the present study, it is an important research topic that will be considered in future studies.

## Figures and Tables

**Table 1:** Crystallization and melting characteristics parameters of different XLPE samples.

XLPE Sample	Crystallization Temperature $T_{C}$ (°C)	Melt Temperature $T_{m}$ (°C)	Crystallinity $X_{C}$ (%)
140 °C pre-crosslinked	89.36	105.14	33.15
150 °C pre-crosslinked	89.70	105.65	33.08
160 °C pre-crosslinked	90.04	106.16	32.85
Normal crosslinking (180 °C)	90.89	106.67	36.52

**Table 2:** Tensile strength and elongation at break of XLPE specimens.

XLPE Sample	Tensile Strength (MPa)	Elongation at Break (%)
140 °C pre-crosslinked	20.08	551.05
150 °C pre-crosslinked	22.52	525.01
160 °C pre-crosslinked	22.77	515.05
Normal crosslinking (180 °C)	24.54	506.48

**Table 3:** AC/DC characteristic breakdown field strength of XLPE samples with different pre-crosslinking degrees.

XLPE Sample	DC Breakdown Field Strength (kV/mm)	AC Breakdown Field Strength (kV/mm)	DC Breakdown Field Strength Reduction (%)	AC Breakdown Field Strength Reduction (%)
140 °C pre-crosslinked	343.1	93.48	9.4	7.6
150 °C pre-crosslinked	357.5	95.25	5.6	5.9
160 °C pre-crosslinked	362.8	99.57	4.2	1.6
Normal crosslinking (180 °C)	378.9	101.20	-	-

## Data Availability

The original contributions presented in this study are included in the article. Further inquiries can be directed to the corresponding author.
